# Digital Smoking Cessation Preferences of Predominately Low-Income and Latino Residents of the San Joaquin Valley in California: Qualitative Study

**DOI:** 10.2196/74105

**Published:** 2025-11-10

**Authors:** Karla D Llanes, Maya Vijayaraghavan, Sara Schneider, Pamela M Ling, Evi Hernandez, Paul Brunetta, Anna V Song, Arturo Durazo

**Affiliations:** 1 Center for Tobacco Control Research and Education, University of California San Francisco, San Francisco, CA, United States; 2 Pharmaceutical Sciences, School of Pharmacy, The University of Texas at El Paso, El Paso, TX, United States; 3 Smoking Cessation Leadership Center, Division of General Internal Medicine, University of California San Francisco, 490 Illinois Street #83C, San Francisco, CA, 94158, United States, 1 628-206-6959; 4 Division of General Internal Medicine, University of California San Francisco, San Francisco, CA, United States; 5 Nicotine and Cannabis Policy Center, Health Sciences Research Institute, University of California Merced, Merced, CA, United States; 6 California Health Collaborative, Fresno, CA, United States; 7 Fontana Tobacco Treatment Center, University of California San Francisco, San Francisco, CA, United States; 8 Psychological Sciences, School of Social Sciences, Humanities and Arts, University of California Merced, Merced, CA, United States; 9 Public Health, School of Social Sciences, Humanities and Arts, University of California Merced, Merced, CA, United States

**Keywords:** mHealth, digital health, smoking cessation, e-cigarette use, smoking, Hispanic, Latino, mobile phone

## Abstract

**Background:**

Although rates of tobacco use in California have declined overall, adults in the San Joaquin Valley (SJV), particularly Hispanic or Latinos (“Latinos”), have disproportionately high rates of tobacco use, tobacco-related illness, and mortality. Residents of the SJV also have limited access to cessation support services and need accessible, nonclinical alternatives. Given high smartphone use rates among Latinos and residents of rural communities, digital health tools may present an accessible approach to expand cessation support.

**Objective:**

This study explored tobacco use behaviors, cessation experiences, and views about digital cessation tools for tobacco cessation among SJV residents. The secondary objective was to assess the appeal, usability, and necessary adaptations of 2 existing digital smoking cessation tools—a smoking cessation app and a social media–based cessation intervention.

**Methods:**

Through an SJV-based academic-community partnership, we recruited 29 predominantly Latino adults who reported current smoking. We conducted 4 focus group discussions to explore tobacco use and cessation experiences and preferences for smoking cessation tools: 1 in-person in English, 1 online in English, and 2 online in Spanish. Subsequently, 9 participants from the focus group discussions completed individual, in-depth interviews where they viewed videos describing 2 digital smoking cessation tools—a cessation app and a social media cessation intervention—to assess their appeal and usability. Focus groups and interviews were recorded, transcribed, and analyzed to identify themes.

**Results:**

Overall, 82.1% (23/28) had made a quit attempt in the past year, and most intended to quit smoking in the next 6 months, with 11.1% (3/27) never expecting to quit. Most participants were motivated to quit despite experiencing barriers, and they emphasized the need for culturally tailored digital cessation tools to help overcome the barriers to quitting smoking. They preferred interventions that integrated culturally relevant content reflecting lived experiences, featured language-concordant communications, and provided social supports, such as chat rooms for peer connection. Participants reported polyuse of tobacco with other substances, including cannabis, which may need to be addressed when delivering smoking cessation interventions. While participants appreciated the app’s private interface and comprehensive curriculum, they preferred the social media–based program for its engaging design, despite privacy concerns. Preferences for specific interventions varied by age and digital literacy. Participants also expressed preference for material rewards to incentivize the use of digital health tools to quit smoking.

**Conclusions:**

This sample of predominantly Latino adults from the SJV expressed favorable interest in digital cessation support, yet existing tools require adaptation to improve cultural relevance, accessibility, usability, and privacy concerns. Participants emphasized language-concordant services, representation from people with lived experience, and community-building features. While digital interventions were well received, privacy concerns and digital literacy barriers must be addressed to enhance engagement.

## Introduction

### Background

Cigarette smoking is the leading cause of preventable death in the United States, with more than 490,000 smoking-related deaths each year [1]. Smoking is associated with detrimental effects to the cardiovascular system, respiratory system, and other health risk outcomes [1]. While overall smoking prevalence has declined in the United States, disparities persist across racial and ethnic groups, particularly among those with low socioeconomic status [2]. For instance, smoking rates are lowest among Asian (5%) and Latino (8%) adults [2] and highest among American Indian Alaska Native (AIAN; 19.3%) adults [2]. However, aggregate rates for Latino adults mark variations. For example, Cuban and Puerto Rican adults report some of the highest rates among Latinos (up to 30%) [3]. African American [4] and Latino adults are heavily targeted in the sale of menthol cigarettes, with a higher density of smoke shops that sell menthol cigarettes in African American or Black and Latino neighborhoods [5,6]. For Latinos, the stress of acculturation to US culture [7] and experiencing discrimination increases the risk of smoking [8,9]. These intersecting factors contribute to tobacco-related disparities across racial or ethnic groups.

While California is recognized as a national leader in tobacco control, with some of the most comprehensive policies and lowest smoking rates in the United States, rural regions maintain disproportionately higher smoking rates [10]. California’s San Joaquin Valley (SJV), a predominantly rural and agricultural region, exemplifies this disparity, with a tobacco use prevalence of 15.6% in 2019 [11], exceeding the state average of 11.4% in 2022 [1]. Home to more than 4 million people across 8 counties (Stanislaus, San Joaquin, Merced, Madera, Fresno, Kings, Tulare, and Kern) [12], SJV includes many structurally marginalized and under-resourced communities. Nearly 45% of SJV residents identify as Latino [12], and 70% of immigrant residents originate from Central America and Mexico [13]. Latinos in California experience a disproportionate burden from tobacco use, with 1.2 million Latinos reporting current use of tobacco, second to Whites at 1.4 million, followed by 0.3 million African Americans and less than 0.1 million AIANs [11].

The SJV region is considered a medical desert, with severe shortages of health care providers, limiting access to health care, specialty services, and tobacco cessation resources. Low literacy skills and limited English proficiency [13] also make it more challenging to navigate health care systems and access cessation support. The SJV also has one of the highest poverty rates in California, with 20% of residents in most counties living below the federal poverty level, compared with 12% statewide [12].

Although Latinos in California attempt to quit smoking at higher rates than the general population (56.5% vs 55.1%), they are less likely to use evidence-based cessation treatment such as nicotine replacement therapy (NRT) and receive cessation advice from health care providers [14]. In 2023, of the 21,500 people who used the free cessation program known as Kick It California, which includes the state Quitline, text, web, and chat support, only 21.1% identified as Latino, while 37.7% identified as non-Hispanic White [10]. Latinos face disparities in access to health care and health insurance [14], and 46.7% of Latino adults who smoked were advised to quit versus 49.9% of all California adults [10], which may contribute to underutilization of cessation supports. Other reasons for not using cessation supports include misperceptions about the addictiveness of NRT [15], hesitation to use support because of the cultural belief that using willpower is a way to take personal responsibility for quitting [16], or using both cigarettes and e-cigarettes (8.1% of Latinos use both vs 8.8% non-Hispanic White) [17], which might make it more challenging to quit [18]. Given these disparities, there is an urgent need for culturally responsive and affirming smoking cessation interventions for Latinos in the SJV. Expanding cessation resources beyond traditional clinical settings—particularly through digital health solutions—may help address gaps in cessation services for Latinos who smoke and face significant barriers to treatment.

While expanding access to health care is critical for increasing cessation treatment utilization, many Latino adults who smoke face persistent barriers such as lack of insurance, limited access to health care providers, and language barriers. These challenges are exacerbated in rural regions such as California’s SJV, where access to health care and cessation services is highly limited. Racial and ethnic minorities in rural areas have limited resources to use health care services [19]. Compared with non-Hispanic Whites (15%), 23.1% of Latinos, 24.5% of non-Hispanic Blacks, and 19.1% of AIANs [1,2] could not see a physician due to elevated cost [19]. Digital cessation tools offer a promising and cost-effective alternative, providing a scalable approach to reaching those who may otherwise lack access to cessation support.

Latinos in the United States have among the highest rates of smartphone adoption (93%) [20]—often using smartphone devices as their primary means of web-based access—compared with 91% of non-Latino Whites. In addition, 95% of Latinos use the web [21] and 84% [22] engage with social media platforms, making digital health interventions a viable strategy for smoking cessation [23-25]. A systematic review of 60 studies found that digital interventions, including mobile apps, text messaging, and web-based programs, significantly increased smoking abstinence compared with printed materials for adult smokers (risk ratios=1.32 for prolonged abstinence and 1.14 for point prevalence abstinence) [26]. These findings highlight the potential of leveraging mobile and social media–based interventions to improve cessation support among Latinos, particularly in hard-to-reach, rural communities such as the SJV.

Social media interventions may also enhance engagement by leveraging existing social networks. For example, the Tobacco Status Project, a 90-day Facebook smoking cessation intervention, delivered evidence-based counseling to young adults in group meetings on Facebook [27]. Social media interventions have been studied in national samples of young adults [27-30] and can be adapted to users’ preferences. Social media–based interventions, whether delivered through existing platforms such as Facebook, Instagram, and WhatsApp, or through specialized platforms, have been shown to be efficacious in increasing abstinence compared with control conditions [31].

Smoking cessation smartphone apps when used in combination with pharmacotherapy may also be an effective aid [32]. A 2023 meta-analysis of randomized controlled trials compared continuous smoking abstinence rates between young adults receiving smartphone text cessation support messages and those receiving only self-help materials or referrals to the Quitline (ie, controls) and found an overall increase in abstinence among those receiving SMS text messaging interventions [33]. The subgroup analysis (*k*=5) showed that continuous abstinence rates were higher in the SMS text messaging interventions than in control groups at 1-month, 3-month, and 6-month follow-ups (relative risks=1.90, 1.64, and 1.35; *P*<.05; respectively); only 2 apps reported continuous abstinence outcomes [33]. Since most apps have not been evaluated in clinical trials or published in scientific literature, it makes determining the efficacy of cessation apps largely inconclusive [33-35]. A 2021 search in the iOS App Store and Android App Store generated 228 apps for smoking cessation, and evaluations of each app revealed that very few apps are evidence-based, and 53% were inaccessible due to the financial costs of using the app after downloading [34]. However, 25% of the apps were available in Spanish, signaling potential interest in Spanish language apps [34]; however, we are not aware of any apps developed specifically for lower-income Latino adults.

The high prevalence of tobacco use, health care shortages, economic hardship, and linguistic barriers creates significant obstacles to smoking cessation in the SJV, particularly for Latino residents. Without accessible, culturally tailored cessation interventions, Latinos in the region remain at heightened risk for tobacco-related illnesses [36]. Although the adoption of digital technology is growing and digital cessation interventions may address these health disparities, little is known whether SJV residents would be interested in using digital cessation interventions to assist with smoking cessation. This study aimed to explore tobacco use behaviors, cessation experiences, and views about digital cessation tools for tobacco cessation among SJV residents.

### Theoretical Frameworks

To guide this study, we applied a widely used framework for identifying behavioral determinants across individual, social, and environmental contexts, the Capability, Opportunity, Motivation–Behavior (COM-B) model [37]. The COM-B model posits that behavior is the result of interactions between individual capability (psychological and physical), opportunity (social and environmental), and motivation [37]. The model’s 3 components interact to either enable or constrain behavior and can serve to inform intervention design and health communications [37]. In this study, the COM-B model was used to identify barriers to and facilitators of quitting smoking and engaging with mobile health platforms.

We also drew from constructs from behavioral change theories to contextualize our secondary objective, evaluating the acceptability and usability of 2 digital cessation tools. For example, the Health Belief Model informed our prompts about perceived risks of smoking and benefits of quitting [38]. In addition, 2 smoking cessation tools were presented to participants—1 mobile app and 1 social media–based cessation intervention—both developed from a combination of these behavioral models and evidence-based cessation strategies. For example, Social Cognitive Theory, we considered constructs relevant to digital interventions when presenting app features (eg, self-regulation, self-efficacy, and model learning) [39]. Both digital tools incorporated social support that can help people model behaviors to avoid smoking and teaching self-regulation. The secondary aim of our study was to explore people’s perceptions of these digital tools, particularly their appeal, usability, and suggested adaptations.

## Methods

### Study Design

In partnership with the California Health Collaborative (CHC), a leading, nonprofit community-based organization in the SJV, we recruited 29 adults from the SJV to participate in the focus group sessions. Coauthor EH, CHC’s senior director of programs for the past 22 years, played a key role in conducting the study. He led participant recruitment across the different counties in the SJV by leveraging his connections with community organizations, which helped identify interested participants. Participants also used word of mouth to let people they know about the study. These strategies allowed us to fulfill our recruitment goals. Eligible participants were aged 18 years or older who reported cigarette smoking in the past 30 days. Participants did not know the researchers prior to the study. We developed focus groups and semistructured interview guides with input from CHC, the study investigators, and undergraduate students proficient in Spanish.

### Data Collection Procedures

#### Brief Survey

We first invited participants to complete a brief survey, available in English or Spanish, which collected demographic characteristics and smoking behaviors. Measures included age, gender identity, race and ethnicity, preferred language and English proficiency, tobacco use history and current behavior, time to first cigarette, quitting intentions, and quitting experiences [40]. To assess financial insecurity, we asked participants whether they were able to save money each month (yes/no), had 3 months of savings to cover an expense, and owned a home. Following the survey, participants were invited to take part in a focus group.

#### Focus Groups

We conducted focus groups using an adapted discussion guide from existing studies to explore participants’ tobacco use, experiences with quit attempts, motivation for quitting, prior use of cessation aids, and resources. We also elicited interest and desired features of digital cessation tools. The focus group discussion used open-ended questions to allow participants to provide their general interest and desired features of digital cessation tools. Each discussion session lasted approximately an hour and began with a brief explanation of the study purpose, ground rules, and an icebreaker. Using open-ended questions, we encouraged participants to discuss smoking and experiences with tobacco use, motivation and experiences with quitting, and their views about resources and cessation aids, including web-based apps to quit smoking.

#### Individual Interviews

Self-selected participants who completed a focus group were subsequently invited to participate in an individual interview. Each 1-hour interview included the presentation of two 10‐15-minute demonstration videos: one describing an app to quit smoking called “MO” [24], and the other presenting a vaping cessation group intervention on Instagram called “Quit the Hit” [25]. The original demonstration videos were created in English by the developers of each tool (PB for the “MO” and PML for “Quit the Hit”). Spanish language versions were translated and narrated by AD (MO) and KDL (Quit the Hit). After viewing each video, participants were interviewed (KDL and SS) about their general impressions, including what they liked and disliked about each tool, and their preferences for one, both, or neither. During the interviews, participants identified additional appealing and unappealing features they might not have generated on their own during the focus groups.

*MO:* The MO app for smoking cessation creates an individual profile, tracks smoking, and suggests activities to help users prepare to quit [41]. It includes gamification awarding points for desired behavior, provides badges to recognize quitting milestones, offers daily check-ins, social chat, and video stories to support motivation [41]. The MO app was developed using the US Clinical Practice guidelines, including the use of the 5 As framework (ie, ask, advise, assess, assist, and arrange) and cognitive behavioral therapy techniques [41].

*Quit the Hit*: Quit the Hit intervention is a person-centered 5-week social media cessation intervention delivered over Instagram [25], adapted from a Facebook smoking cessation program [28,29,42] that increased abstinence at the end of treatment (3 months) compared with referral to a smoking cessation website [27]. Participants join support groups moderated by a cessation counselor with weekday posts supporting preparation, skill building, a group quit date, and social supports to quit. The intervention is for a person ready to make a behavior change, developed using the US Clinical Practice Guidelines for smoking cessation and Social Cognitive Theory. Similarly to the MO app, Quit the Hit uses user-centered, evidence-based intervention content adapted from cognitive behavioral theory and motivational interviewing (eg, increasing self-efficacy and commitment to make a change). Participants were informed that Quit the Hit was adapted from a smoking cessation intervention and could be adapted to address other tobacco use.

### Procedures

#### Study Procedures for the Focus Groups and Semistructured Interviews

We obtained informed consent prior to conducting all study procedures. AD led the initial round of informed consent in both English and Spanish before administering the survey. All participants read and provided informed consent before proceeding with the survey, focus groups, and interviews. Participants were identified through a self-generated study ID number to protect their confidentiality.

#### Survey Administration

First, participants completed the brief survey assessing their tobacco history, interest in quitting, and past methods used for smoking cessation. Participants in the web-based focus groups completed the brief surveys on Qualtrics prior to the focus group session. For those who had not completed the survey in advance, a few minutes were set aside at the beginning of the focus group session to do so. Participants in the in-person focus group completed a paper version of the survey before the focus group session began.

#### Focus Group Protocol: Needs Assessment for Digital Smoking Cessation Support

After completing the survey, 29 participants participated in a focus group discussion about their tobacco use behaviors, cessation experiences, and views about digital cessation tools for tobacco cessation. One participant did not want to fill out information about their drug use or participate in the focus group because of legal concerns. Focus groups took place in-person or via Zoom (Zoom Communications, Inc.), in English or Spanish, depending on the participants’ preference. Three focus groups were conducted on the web (2 in Spanish and 1 in English), and 1 focus group was conducted in person (in English). There were 8 participants in each of the 2 web-based Spanish focus groups, 5 in the web-based English focus group, and 8 in the in-person English focus group. The focus groups lasted 60‐90 minutes and were led by moderators (AD and KDL) accompanied by notetakers. Authors AD and KDL, who are proficient in English and Spanish, moderated the focus group discussions. The focus groups were conducted from March 2023 to April 2023. An external professional transcription company (Focus Forward) transcribed the English and Spanish audio of focus group discussions.

#### Semistructured Interview Protocol: Appeal and Usability of Digital Smoking Cessation Tools

We reached out to all 29 participants who participated in the focus group and invited them to participate in a follow-up semistructured interview. In total, only 9 were available and agreed to participate in the 1-hour follow-up interview. Three (1 female and 2 males) interviews were conducted in English and 6 (4 females and 2 males) were conducted in Spanish. KDL and SS conducted the semistructured interviews between November 2023 and February 2024. An external professional transcription company (GMR Transcription) transcribed the audio of interviews.

### Data Analysis

Audio files were transcribed verbatim and translated from Spanish to English and uploaded to ATLAS.ti (version 24; Scientific Software Development GmbH). Spanish-speaking researchers (AD and KDL) read the Spanish-transcribed and translated files to identify errors in the transcription process. Two coders (KDL and SS) read each transcript, coded them, and identified themes. We used thematic analysis (inductive approach) to identify individuals’ needs for smoking cessation digital tools to facilitate their quitting process [43,44]. The codebook was refined as new themes were identified during the coding process. Discrepancies in coding were discussed and resolved by consensus, including discussion with a third coder (AD), if necessary.

### Positionality

We paid particular attention to ensure that participants fully understood the nature of the research and their rights, given potential language barriers and vulnerabilities. We began each focus group with icebreakers and an introduction of ourselves as researchers, along with an explanation of the study’s purpose. We also acknowledge our own positionalities as researchers and how they may have influenced the research process. AD is a health communication researcher and is a second-generation Mexican American Californian who grew up with Spanish as his first language, and KDL is a bilingual (ie, first language was Spanish), Latina public health postdoctoral scholar with roots in the Southwest. Our shared cultural and linguistic backgrounds contributed to trust-building with participants, particularly in communications requiring sensitivity to language access and cessation support experiences among racial or ethnic minority and Spanish-speaking individuals. The bilingual team conducting the focus groups and interviews ensured that information was not lost in translation because participants freely expressed themselves in their preferred language. We reviewed the focus group and interview guides with research interns who brought lived experience as SJV residents. In addition, to ensure cultural and linguistic appropriateness, our community partner and coauthor, EH, who grew up in the region, reviewed and provided feedback on the Spanish translations of the consent form, survey, and focus group guide. This partnership helped ensure that research materials were accessible and culturally relevant for SJV residents.

### Ethical Considerations

This study was reviewed and approved by the University of California, San Francisco (IRB 22‐37978) and University of California, Merced (IRB UCM2022-119) institutional review boards. All participants read and provided informed consent on Zoom or in person. Researchers reassured participants of confidentiality. Participants were also offered a printed or electronic copy of the informed consent form. The researchers deidentified surveys, focus groups, and interviews by using a self-generated ID number for each participant. We used the COREQ (Consolidated Criteria for Reporting Qualitative Research) to describe and present the research findings to fit standard qualitative reporting. We attest that we did not use generative AI to write any portion of the manuscript or for data analyses. Participants each received a US $50 gift card for participating in the focus group and an additional US $50 gift card for participating in an in-depth interview.

## Results

### Sample Characteristics

Although 29 participants participated in the focus groups, not all completed all survey items. Participants were permitted to skip questions they did not want to answer. The majority of participants were Latino (24/27, 88.9%), 51.9% (14/27) self-identified as female, and mean average age was 41 years (SD 14.04, range 21‐77). Most (14/26, 53.8%) preferred Spanish language, 38.4% (10/26) reported limited English proficiency, and 63% (17/27) reported that they were unable to save money each month (Table 1). Those who preferred Spanish were predominantly from Madera County, older, preferred smoking cigarettes, and were less comfortable with technology. Focus group participants from Fresno County were younger, English-speaking, primarily used e-cigarettes, and were comfortable with technology. Participants from Merced County were older, preferred English language, most smoked cigarettes, and the focus group was conducted in person. Overall, 82.1% (23/28) had made a quit attempt in the past year, and most intended to quit smoking in a month to 6 months, with 11.1% (3/27) never expecting to quit.

**Table 1.** Demographic characteristics of participants in focus group discussions (n=29)^a^.

Characteristics	2 Focus groups from Madera (n=16)	1 Focus group from Fresno (n=5)	1 Focus group from Merced (n=8)	Overall (N*=*29)
Age (years), median (IQR)	46^b^ (15)	25 (6.5)	41 (18)	41 (22)
Sex, n (%)				
Female	10 (71.4)	4 (80)	0 (0)	14 (51.9)
Male	4 (28.6)	1 (20)	8 (100)	13 (48.1)
Race and ethnicity, n (%)				
Non-Hispanic White	0 (0)	0 (0)	3 (37.5)	3 (11.1)
Hispanic/Latino^c^	14 (100)	5 (100)	5 (62.5)	24 (88.9)
Preferred language, n (%)				
English	1 (7.1)	5 (100)	6 (85.7)	12 (46.2)
Spanish	13 (92.9)	0 (0)	1 (14.3)	14 (53.8)
English proficiency (English language preference), n (%)				
Very well	0 (0)	5 (100)	7 (100)	12 (46.2)
Well	4 (28.6)	0 (0)	0 (0)	4 (15.4)
Not well	7 (50)	0 (0)	0 (0)	7 (26.9)
Not at all	3 (21.4)	0 (0)	0 (0)	3 (11.5)
Years smoked, mean (SD)	18.4 (14)	3.2 (1.4)	12.4 (11.8)	14.0 (13.1)
Cigarettes smoked per day, mean (SD)	2.1 (2.8)	2.8 (0.8)	13.5 (8.5)	5.5 (8.1)
Time to first cigarette use after waking up, n (%)				
Within 5 minutes	1 (6.7)	1 (20)	3 (37.5)	5 (17.9)
6‐30 minutes	1 (6.7)	1(20)	2 (25)	4 (14.3)
After 60 minutes	10 (66.7)	3 (60)	2 (25)	15 (53.6)
Question is unclear	3 (20)	0 (0)	1 (12.5)	4 (14.3)
Voluntary quit smoking for 24 hours in past year, n (%)				
No	2 (13.3)	1 (20)	2 (25)	5 (17.9)
Yes	13 (86.7)	4 (80)	6 (75)	23 (82.1)
Quitting intentions, n (%)				
Never expect to quit	1 (7.1)	1 (20)	1 (12.5)	3 (11.1)
Will quit in the next month	5 (35.7)	2 (40)	2 (25)	9 (33.3)
May quit in the next 6 months	1 (7.1)	1 (20)	4 (50)	6 (22.2)
Will quit in the next 6 months	7 (50)	1 (20)	1 (12.5)	9 (33.3)
Ability to save each month, n (%)				
No	10 (71.4)	0 (0)	7 (87.5)	17(63)
Yes	4 (28.6)	5 (100)	1 (12.5)	10 (37)
3 months savings to cover expenses, n (%)				
No	12 (85.7)	3 (60)	8 (100)	23 (85.2)
Yes	2 (14.3)	2 (40)	0 (0)	4 (14.8)
Own home, n (%)				
No	6 (42.9)	5 (100)	8 (100)	19 (70.4)
Yes	8 (57.1)	0 (0)	0 (0)	8 (29.6)

a Percentages for all variables include only those participants who provided a response to survey items. Responses are complete for 26 participants. Four participants skipped questions resulting in missing data for all the variables.

b Two participants did not report their age.

c Hispanic/Latino category included individuals selecting any race or not selecting a race category.

### Focus Groups

#### Smoking and Vaping Initiation and Current Use

Participants reported initiating smoking at a young age, with many starting between 9 and 20 years. Some began with materials other than tobacco, such as smoking lawn grass. Family influences played a significant role in cigarette-smoking behaviors, with multigenerational influences being common.

*I learned from my dad...I would steal his cigars, I would even get drunk with him...from then on, I started to want to buy cigarettes. I used to make corn leaves, which came from him, toasted tobacco in a corn leaf*.

[63-year-old, male, Madera]

Social settings also contributed to the initiation and continuation of tobacco use, especially use at parties and while drinking alcohol.

*Definitely when you're drinking alcohol, a lot of friends just drinking and a couple of them have it, you just decide to experiment with it, but then you decide to go off and buy your own, even when you're not drinking, and it becomes more of an addiction.*

[Age, sex, and location not disclosed]

Using tobacco to cope with stress was a critical factor, contributing to sustained use and diminished motivation to quit.

*[E]very time you have anxiety or are stressed, you have a lot of work, the kids are yelling at each other, or whatever, you go out at night, and you take [a cigarette].*

[39-year-old, female, Madera]

### Motivations to Quit Smoking or Vaping

Many in the older Spanish-speaking group were parents, and their motivation to quit was their children or family. For the younger English-speaking group, having children was a reason to quit in the future but not currently. Participants described being concerned about their health. Illnesses made them more aware of their overall health and motivated them to quit or reduce smoking. One participant got COVID-19 disease, another had asthma, and a third was worried about developing cancer.

*So, like, I got COVID and I was just really, really scared because that was the first time I had gotten it, and I was like, “Maybe—yeah, I think I just have to stop doing everything altogether.”*

[24-year-old, female, Fresno]

Tobacco regulation efforts such as flavor bans or price increases motivated some individuals to quit smoking or vaping. Flavors enticed many younger participants to initiate vaping, and a participant reported that she smoked less because of the flavor ban.

*That new law that got passed with the flavored vapes has made things super inaccessible and super hard to find, so that has been one reason why I have not been smoking too much lately.*

[24-year-old, female, Fresno]

Participants tried different methods to quit smoking and vaping. Only a few participants had used NRT, some when hospitalized, which had helped them to temporarily quit. Most had tried to quit on their own (ie, cold turkey), and one participant interpreted ongoing use of NRT as a reason not to use it.

*I've been trying to quit cold turkey. I don’t really feel like the gum or anything like that works too well, and my brother did actually quit, and he used the gum. It was hard for him to even stop using the gum because it still has the nicotine in it. His recommendation to me was to quit cold turkey*.

[Age, sex, and location not disclosed]

A few participants used cannabis to cope with nicotine withdrawal symptoms.

*Smoking marijuana at night to get me to sleep was actually pretty helpful because I would go to sleep, with cold sweats, not having the nicotine in my system.*

[Age, sex, and location not disclosed]

Participants described that tobacco and other substance use was common, where some participants substituted other substances (eg, cannabis) for smoking. One participant found that decreasing the use of other substances decreased their cigarette consumption.

*Crystal [methamphetamine] and cigarettes went hand in hand. After you use one, I mean, you're gonna want the other, a finishing, topping off, type deal...I’d slow down, and then I stopped doing crystal, and then I kinda slowed down smoking.*

[46-year-old male, Merced]

Another individual switched from combustible cigarettes to e-cigarettes to quit smoking but found that he instead increased nicotine use.

*I did try to quit, but this was when the vape stuff started coming out, those little box mods. I found out that it made me crave tobacco even more. So, my smoking habit doubled from the time that I started vaping.*

[Age not disclosed, male, Merced]

### Recommendations for Digital Cessation Tools

Many of the Spanish-speaking participants in the focus groups felt that what was available did not meet the specific needs, challenges, or preferences of other members in their community. Participants discussed that smoking cessation tools should be not only linguistically appropriate but also include the lived experience of someone who understands their culture. Offering services in Spanish alone was not sufficient to be culturally appropriate.

*People who really correctly understand the words that we’re saying, who understand and comprehend us. If they know the culture, maybe they know a reason why we seek refuge in cigarettes...Maybe they could understand us and help us a little more.*

[46-year-old, female, Madera]

In addition to information on quitting, participants felt that instructional materials to increase awareness of the dangers of secondhand smoke should be incorporated.

*I think it’s really important for all of us smokers to be aware that secondhand smoke is harmful, especially for our immediate family, our spouse, our kids, even our neighbors or coworkers*.

[54-year-old, male, Madera]

When asked for general recommendations for digital cessation tools, participants expressed interest in social connections.

*Just the fact of being able to kind of communicate with people that are dealing with the same issues as you is nice.*

[24-year-old, female, Fresno]

Younger participants from Fresno also expressed interest in “more real-life examples instead of just a big X on a vape pen” [36-year-old, female, Fresno] and something easy to navigate.

*Something that’s user friendly. Something that doesn’t require too many clicks. There’s nothing worse than downloading an app, or opening a website, and “Oh, you have to do this step, and this step.*

[24-year-old, female, Fresno]

Some individuals expressed interest in daily digital posts that they could relate to.

*I think something like an app designed to hook you in. I know a lot of people open up these like word of the day, something that’ll cause you to think, “Okay. Am I feeling that? I think that would be a good way to help people stop smoking, if you have a day to day, like, “Oh, let me check the app and see if I can relate to it today.””*

[25-year-old, female, Fresno]

### Semistructured Interviews on Perspectives of the MO App and Quit the Hit

#### Appealing Features of the MO Smoking Cessation App

Participants liked a feature of the MO app, which was a “crave or urge button” that connected to immediate cessation support on demand. They also liked being able to track their own data on their use behaviors on the app.

*I also like the urge button where the photo was pulled...when you’re kind of struggling in that moment, it gives you immediate assistance*.

[25-year-old, female, Fresno]

Participants found relapse planning features of the app, including tips to modify their environment or rituals to avoid smoking cues that increase cravings (eg, avoiding the usual route to work where they buy cigarettes, rearranging furniture), to be helpful.

*It changes your route. If you go to the store to buy a cigarette, it’ll try to change your route. It’s got ways that help you quit smoking. It figures your [routine]. Basically, trying to prevent you from smoking.*

[46-year-old, male, Merced]

Some individuals preferred the app because it offered privacy for cessation support, which they preferred to interacting over social media.

*It kind of reminded me of a journal.*

[24-year-old, female, Fresno]

### Appealing Features of the “Quit the Hit” Social Media Support Groups

Participants liked the interactive engaging content and opportunity for social and peer support with people with shared experience:

*It’s cool, comfortable, and more interactive with other people; it feels more human and more real, sharing the challenge because it feels like a challenge.*

[39-year-old, female, Madera]

Other appealing features of Quit The Hit were the availability and accessibility of a peer counselor, similar to other in-person models of substance use treatment such as Alcoholics Anonymous or Narcotics Anonymous.

*The main thing is that week by week a counselor will talk to us to give us points of view that would be good to help us that week.*

[46-year-old, female, Madera]

Participants liked the idea of interacting with other members of their group when they needed it as social support and to hold themselves accountable.

*I liked the fact that the other members of the group motivate each other and give you advice. They help you not to get discouraged if you fail, don't stop, start again.*

[Age not disclosed, female, Madera]

Participants also liked that the program was structured over 5 weeks, perceiving that the time allotted allowed substantial progress in behavior change at their own pace.

*They give you time, let’s say the message you are sending me is that in five weeks I can make a big change.*

[46-year-old, female, Madera]

### Unappealing Features of MO and Quit the Hit Digital Interventions

A few participants expressed dissatisfaction with both interventions, describing practical inconveniences in their daily routines. For instance, a participant highlighted difficulties engaging with interventions while at work.

*For some people it’s functional, but...I couldn't be tracking or marking that kind of stuff because I work with clients.*

[57-year-old, female, Madera]

Some participants reported technology-related concerns with an app such as a need for extra storage space on their device, or the need for a web-based connection or a charged battery. Other participants were uninterested in social media interventions, preferring “person to person contact” [57-year-old, female, Madera]. Others expressed concerns about privacy.

*When you’re adding and kind of joining with other people, my personal information is also out there. So, for some people, they like creating that community and making friends. But also, I like to keep my stuff separate.*

[24-year-old, female, Fresno]

Some participants felt that the support should be anonymous to avoid stigma.

*The negative stigma that comes from addiction...Yeah, your business. It’s supposed to be anonymous like N[arcotics] A[nonymous]. Nobody talks about it. There’s no record of it. So, I would probably go off of social media.*

[Age not disclosed, male, Merced]

Other participants who did not currently use social media reported not wanting to join social media simply to access a support group.

### Recommendations for Culturally Tailored Digital Interventions

Participants expressed interest in several features that could apply broadly to digital cessation tools. For example, participants expressed interest in a chance to earn prizes, money, or other tangible compensation as opposed to points.

*Coupons, restaurants that are near them, maybe coupons for the grocery store or free Pepsis, free gum. I don’t know, some kind of coupons for something.*

[46-year-old, male, Merced]

Participants expressed a need for digital tools to be visually appealing and attention-grabbing. They also suggested materials to discuss tobacco use and vaping within the context of other life stressors and other addictions, including substance use.

*Addictions can be to tobacco, drugs, food, or any other substance, and almost all of them have the same components here in the brain, and they are treated in the same way. Psychologists or psychiatrists treat it in the same way and with very similar medications.*

[54-year-old, female, Madera]

Participants reported that linking treatment for tobacco use with other substance use could help prioritize the issue of tobacco use, particularly in SJV counties where counseling and support services are scarce.

*Like I said, other chemicals and everything else, because it’s not really informative over here, especially in our location where it’s at with the behavioral health aspect. You could probably partner up with them and then get professional opinions or have someone on standby, when people have a burning desire or something, if you were to go out with the different aspects of every chemical out there, not just tobacco, because tobacco’s really not a priority right now for most of us.*

[Age not disclosed, male, Merced]

They expressed interest in linkages to other related interventions such as acupuncture or games to keep busy and cope with stressors.

*I would still add relaxation techniques, breathing techniques. I have been to acupuncture, and it helps a lot with anxiety and stress.*

[46-year-old, female, Madera]

## Discussion

### Principal Findings

Our research highlights the need for tobacco cessation tools for people living in economically challenged rural areas, such as the SJV. Our study indicated that both older and younger predominantly Latino adults who smoked or vaped nicotine were amenable to digital cessation tools, particularly when these interventions were delivered in a culturally affirming way that included not only language concordance but also relevant lived experiences. Consistent with past studies [14,45], participants smoked or vaped as a coping response to stressors [46], many did not use pharmacotherapy to quit, and most attempted to quit on their own. Latinos who smoke may hold misperceptions about the addictiveness of NRT [15]. In this study, SJV residents were skeptical of the use of NRT and believed that NRT may be as addictive as other nicotine products. This suggests a need for tailored programs to support evidence-based counseling and education about pharmacotherapy to improve cessation outcomes [47].

Older Spanish-speaking participants preferred culturally tailored tobacco cessation services. Participants emphasized the need for counselors or messages to express empathy toward their particular circumstances, as well as needs for relatable peer support and personalized approaches such as individualized text messaging in Spanish to increase engagement among Latino adults [48].

To overcome these barriers, evidence-based solutions can be integrated into the design and delivery of digital cessation tools. First, digital health interventions delivered or introduced by trained community health workers (CHWs) or *promotoras/es* (Spanish-speaking CHWs) from the SJV might increase buy-in among older, Spanish-speaking adults [49]. CHWs and promotoras/es, as trusted members of the community who speak the same language and understand cultural practices, can be trained to deliver tobacco cessation interventions supported by digital tools [49]. In prior work, lay health worker cessation interventions had superior abstinence rates compared with referrals to a Spanish language Quitline (20.5% vs 8.7%, respectively) [50]. Second, providing culturally tailored, accessible cessation materials (eg, mailed written material) to adults who prefer Spanish educational material improves quit rates [51]. A pilot study evaluating a culturally and linguistically tailored text message intervention combined with NRT found moderate to high levels of acceptability, good engagement measured by the number of text messages participants sent during the 12 weeks of program, and improvements in self-efficacy; this intervention reported a 30% biochemically verified abstinence rate [52].

While older participants expressed openness to using digital interventions, some lacked the familiarity and comfort with social media to engage with these platforms. According to the COM-B model, having the necessary skills (ie, the psychological capability) and the opportunity to use social media if a social media platform is adopted may increase the chance of smoking cessation [37]. Participants’ willingness to use digital interventions suggests that the CHWs and promotoras/es model may be beneficial in increasing comfort with technology. These trusted members of the community could support participants by providing guidance on how to use the digital tools prior to the delivery of intervention content [37]. Alternatively, other social media communications platforms such as WhatsApp may be better suited for residents of the SJV than a downloadable app or Instagram interventions. To address concerns about technology issues, participants who are less familiar with apps or social media platforms must receive training to help them navigate the technology with access to offline resources.

In contrast, younger participants were more receptive to social media–based interventions but emphasized the need for attention-grabbing, user-friendly, and easy-to-navigate tools. Younger participants did not emphasize culturally affirming support as much as older participants but expressed interest in peer supports and the importance of integrating lived experience of quitting. A meta-analysis of 16 randomized controlled trials found that peer support interventions were more effective for smoking cessation (mean RR 1.34, 95% CI 1.11‐1.62) and more effective when peer support had quitting experience (*k*=5 studies) (mean RR1.43, 95% CI 1.17‐1.74) [53]. To increase the use of digital cessation tools, it is important to account for digital literacy, technological issues (eg, internet connectivity or memory or battery life), peer support, and age-appropriate messaging [54,55].

Another key COM-B construct that affects the adoption of cessation tools is opportunity to engage with intervention content [37]. A few participants noted that job constraints would make it difficult to meet the demands of engaging with the cessation intervention content particularly during work hours. The limited availability highlights the need for digital cessation content that offers flexibility, whether delivered via smartphone app or social media platforms.

The motivation construct of COM-B will also affect the adoption of cessation tools [37]. Participants also expressed interest in tangible rewards such as money incentives and reminders. Contingency management is an intervention based on operant conditioning, which delivers contingent monetary rewards based on behavior change (eg, smoking cessation) [56]. A 2019 meta-analysis (k=7) found that mobile phone–delivered contingency management was efficacious for tobacco cessation [56]. Overall, we found that smartphone apps [57] and social media interventions [58] are acceptable digital health tools that could help Latinos quit smoking if tailored to enhance engagement [58]. Incorporating digital tools and health interventions is appealing because health information can reach audiences and messages can be tailored to meet individuals’ personal needs or risks.

Participants reported polyuse of tobacco with other substances, including cannabis and crystal methamphetamine, but neither digital intervention offered treatment for polyuse. Treatment for tobacco and substance use can be integrated to improve substance use outcomes and should be investigated further in this population [59]. Smoking cessation interventions among people with substance use disorders can increase long-term abstinence from all substances by 25% [60]. Participants expressed that there was a dearth of substance use treatment supports in the SJV and suggested tobacco cessation to be integrated with mental health and substance use treatment resources.

### Limitations

Although inclusive of Spanish-speaking Latino participants, these findings are from a small sample (29 participants) from 3 counties (Madera, Kings, and Fresno), recruited through community-based organizations, which might not generalize to other California populations, including the remaining 5 SJV counties. Only 9 participants were presented with videos of 2 prototypes of digital smoking cessation tools via interview rather than directly testing them, and recommendations may differ if the cessation tools were used in real-world settings. User testing among users and nonusers of the app could provide valuable feedback for improving the digital tools. Digital interventions that address stress as a trigger and that integrate pharmacotherapy may be beneficial.

### Strengths

While our study included participants from 3 of the 8 counties that comprise SJV, these counties are broadly reflective of the region’s structural and demographic characteristics. All 3 counties are also considered medically underserved, with persistent health care provider shortages and limited access to smoking cessation services [61]. Our study captured the unique voices from people who are often underrepresented in cessation research, particularly Spanish-speaking Latino individuals in the United States and rural settings. Additional strengths of this study are its successful engagement with Latino residents in underserved, rural communities, leveraging trusted community partners and Spanish-speaking researchers to ensure participants’ comfort and authenticity in responses.

### Conclusions

This study provides insights into digital smoking cessation tool preferences among predominantly low-income and Latino residents of the SJV, a region with urgent need of cessation services. Using a community-engaged approach, we found that participants expressed interest in linguistically appropriate and culturally tailored digital cessation tools featuring empathy toward their circumstances, relatable peer supports, engaging content, easy access to information and support, and social or material rewards. Our findings underscore the need for customizable, culturally affirming digital health interventions for tobacco cessation in the SJV, with a focus on social support and user engagement strategies.

## Supplementary material

10.2196/74105 Checklist 1 COREQ checklist.

## Abbreviations

AIAN: American Indian Alaska Native
CHC: California Health Collaborative
CHW: community health worker
COM-B: Capability, Opportunity, Motivation–Behavior
COREQ: Consolidated Criteria for Reporting Qualitative Research
NRT: nicotine replacement therapy
SJV: San Joaquin Valley
