# Supplementary material for: Digital Smoking Cessation Preferences of Predominately Low-Income and Latino Residents of the San Joaquin Valley in California: Qualitative Study
Source: JMIR Form Res. 2025 Nov 10;9:e74105. doi: 10.2196/74105 (PMC12599980; doi:10.2196/74105)
Supplement: Checklist 1 [file formative-v9-e74105-s001.pdf]

COREQ (COnsolidated criteria for REporting Qualitative research) Checklist

A checklist of items that should be included in reports of qualitative research. You must report the page number in your manuscript where you consider each of the items listed in this checklist. If you have not included this information, either revise your manuscript accordingly before submitting or note N/A.

Topic	Item No.	Guide Questions/Description	Reported on Page No.
**Domain 1: Research team and reflexivity**			
Personal characteristics			
Interviewer/facilitator	1	Which author/s conducted the interview or focus group?	Pages 13-14
Credentials	2	What were the researcher's credentials? E.g. PhD, MD	Pages 13-14
Occupation	3	What was their occupation at the time of the study?	Pages 13-14
Gender	4	Was the researcher male or female?	Pages 13-24
Experience and training	5	What experience or training did the researcher have?	Pages 13-14
Relationship with Participants			
Relationship established	6	Was a relationship established prior to study commencement?	Page 10
Participant knowledge of the interviewer	7	What did the participants know about the researcher? e.g. personal goals, reasons for doing the research	Pages 14-15
Interviewer characteristics	8	What characteristics were reported about the inter viewer/facilitator? e.g. Bias, assumptions, reasons and interests in the research topic	Pages 14-15
**Domain 2: Study design**			
Theoretical framework			
Methodological orientation and Theory	9	What methodological orientation was stated to underpin the study? e.g. grounded theory, discourse analysis, ethnography, phenomenology, content analysis	Pages 9-10
Participant selection			
Sampling	10	How were participants selected? e.g. purposive, convenience, consecutive, snowball	Page 10
Sample size	12	How many participants were in the study?	Page 14
Non-participation	13	How many people refused to participate or dropped out? Reasons?	Page 13
Setting			
Setting of data collection	14	Where was the data collected? e.g. home, clinic, workplace	Page 13
Presence of non-participants	15	Was anyone else present besides the participants and researchers?	Pages 13-14
Description of sample	16	What are the important characteristics of the sample? e.g. demographic data, date	Pages 16-18
Data collection			
Interview guide	17	Were questions, prompts, guides provided by the authors? Was it pilot tested?	N/A. No, guides were not included in the manuscript. Guides were pilot tested (Page 10)
Repeat interviews	18	Were repeat inter views carried out? If yes, how many?	Page 14
Audio/visual recording	19	Did the research use audio or visual recording to collect the data?	Page 14
Field notes	20	Were field notes made during and/or after the inter view or focus	Pages 13-14

		group?	
Duration	21	What was the duration of the inter views or focus group?	Page 11
Data saturation	22	Was data saturation discussed?	Pages 18-25
Transcripts returned	23	Were transcripts returned to participants for comment and/or corrections?	No

Topic	Item No.	Guide Questions/Description	Reported on Page No.
**Domain 3: analysis and findings**			
Data analysis			
Number of data coders	24	How many data coders coded the data?	Page 14
Description of the coding tree	25	Did authors provide a description of the coding tree?	N/A
Derivation of themes	26	Were themes identified in advance or derived from the data?	Page 14
Software	27	What software, if applicable, was used to manage the data?	Page 14
Participant checking	28	Did participants provide feedback on the findings?	N/A. No
Reporting			
Quotations presented	29	Were participant quotations presented to illustrate the themes/findings?<br>Was each quotation identified? e.g. participant number	Pages 18-25
Data and findings consistent	30	Was there consistency between the data presented and the findings?	Pages 18-25
Clarity of major themes	31	Were major themes clearly presented in the findings?	Pages 18-25
Clarity of minor themes	32	Is there a description of diverse cases or discussion of minor themes?	Pages 18-25

Developed from: Tong A, Sainsbury P, Craig J. Consolidated criteria for reporting qualitative research (COREQ): a 32-item checklist for interviews and focus groups. *International Journal for Quality in Health Care*. 2007. Volume 19, Number 6: pp. 349 – 357
